# Response Patterns and Mechanisms of Seed Germination and Mortality of Common Plants in Subalpine Wet Meadows to In Situ Burial

**DOI:** 10.3390/plants14192975

**Published:** 2025-09-25

**Authors:** Suyao Yuan, Haijun Cui, Yuzhen Liu, Weifeng Song, Junbao Yu, Jie Li, Xuyan Zhao, Xiaoyan Wei, Xiaoting Bi, Putao Zhang, Tingting Wang, Jingyuan Pu

**Affiliations:** 1 Yunnan Key Laboratory of Plateau Wetland Conservation, Restoration and Ecological Services, Southwest Forestry University, Kunming 650224, China; 15108880184@163.com (S.Y.); songwf85@swfu.edu.cn (W.S.); jbyu@swfu.edu.cn (J.Y.); 18307870018@126.com (X.W.); arrebol98@126.com (X.B.); 18844282676@163.com (P.Z.); 15087517420@163.com (T.W.); pujingyuan2024@163.com (J.P.); 2 Shangri-La Potatso National Park Bita Lake Plateau Wetland Ecosystem Observation and Research Station of Yunnan Province, Kunming 650224, China; 3 Yunnan Dianchi Wetland Ecosystem National Observation and Research Station, National Forestry and Grassland Administration, Kunming 650224, China; 4 Dianchi Lake Ecosystem Observation and Research Station of Yunnan Province, Kunming 650224, China; 5 Academy of Animal Science and Veterinary Medicine, Qinghai University, Xining 810016, China; liuyzqhu@163.com; 6 Institute of International Rivers and Eco-Security, Yunnan University, Kunming 650091, China; jie_li@ynu.edu.cn; 7 Napahai Provincial Nature Reserve Management and Protection Bureau, Shangri-La 674499, China; zxy778161@163.com

**Keywords:** in situ burial, germination rate, mortality rate, seed size, soil seed bank

## Abstract

The effects of different storage conditions on seed germination and mortality may exhibit species-specific patterns. Burial serves as a natural seed storage mechanism, and its impact on seed germination and mortality holds critical implications for understanding the formation mechanisms of soil seed banks and the restoration of vegetation. Seed size is closely related to storage conditions, as it affects the ease with which seeds penetrate the soil, thereby potentially influencing their germination and mortality responses to those storage conditions. This study used 12 common plant species from a subalpine wet meadow. Employing in situ unheated storage as the control and in situ burial at a 15 cm depth (for seven months) as the experimental treatment, we aimed to explore the effects of burial on seed germination and survival, as well as the underlying mechanisms, in relation to seed size. The results showed the following: (1) Compared with the control, the burial treatment significantly increased the germination rates of four species (burial-promoted germination type), while no significant effect was observed on the germination of the remaining eight species (burial-insensitive germination type); it significantly increased the mortality rate of two species (survival-inhibited type), significantly decreased the mortality rate of four species (survival-promoted type), and had no significant impact on the mortality rate of the remaining six species (survival-insensitive type). (2) Seed size exhibited significant negative correlations with both post-burial germination rates and mortality rates under control conditions, while showing a significant positive correlation with the magnitude of mortality change. The species-specific responses of seed germination and mortality to storage conditions, and their close association with seed size, represent products of long-term plant evolution. This study provides important insights for understanding the mechanisms of soil seed bank formation and offers valuable guidance for vegetation restoration practices.

## 1. Introduction

Seed storage conditions are closely related to seed germination and survival [1,2]. Artificial seed storage methods often simulate the storage process in natural environments. For example, in situ and unheated storage facilities can mimic natural temperature regimes, promote seed vernalization, and are widely used in ecological restoration and seed conservation practices [3,4]. However, there may not exist a universal storage condition that exerts positive effects on seed germination and survival across all species [5]. Owing to significant differences in seed dispersal strategies among species [6], the microenvironments they inhabit are highly heterogeneous, which may lead to pronounced species-specific responses of seed germination and survival to storage conditions [5]. For instance, after maturation, seeds of some species are retained within litter, others are dispersed on the soil surface, while still others become incorporated into the soil and undergo the effects of burial [7,8]. This specificity may reflect the outcome of long-term adaptation of different species to their specific environmental conditions. Therefore, investigating the differential effects of various storage conditions on seed germination and survival among plant species not only contributes to revealing their reproductive adaptation strategies, but also provides valuable guidance for vegetation restoration practices.

As a key event commonly experienced by seeds in their natural habitats, in situ burial profoundly influences seed germination and mortality patterns through the complex microenvironment it creates [9]. Burial influences seed germination and survival by altering key environmental conditions such as light availability, temperature, and moisture levels, all of which are critically linked to these physiological processes [10,11]. The physicochemical properties of the burial soil (e.g., soil pH) and soil microorganisms (particularly pathogenic microorganisms) influence seed dormancy levels and survival status [12,13], thereby affecting plant population regeneration processes and population dynamics [14,15]. Generally, small seeds are more likely to become incorporated into the soil, while large seeds are often restricted to the surface or shallow layers due to physical constraints [16,17]. Following natural disturbances (e.g., animal burrowing, trampling, etc.), small-seeded species often exhibit rapid germination, enabling them to function as pioneer species in community succession processes [18]. This implies that soil burial may be more favorable for the survival and germination of small seeds, whereas it may have no significant effect or even an inhibitory effect on large seeds.

Seed size, as a key functional trait, profoundly influences its germination strategies and environmental response mechanisms [19]. Large seeds typically possess greater nutrient reserves and more developed protective embryonic structures, which enhance their resistance to adversities such as drought and low temperatures [18,20]. In contrast, small seeds exhibit a high embryo-to-seed ratio and limited nutrient reserves. Their germination often relies heavily on environmental cues such as light availability, moisture, and temperature, which make them more responsive to changes in external conditions [21,22]. Exposure to air subjects seeds to intense fluctuations in temperature and significant variations in moisture, which can be detrimental to the long-term survival of small seeds. In contrast, the soil environment provides a buffering effect against these fluctuations in temperature and humidity, offering a more stable microenvironment for small seeds and potentially leading to a significant reduction in their mortality rates.

This study studied the seeds of 12 common plant species from a subalpine wet meadow. Two seed storage conditions were established: one simulating common artificial storage with natural temperature fluctuations (in situ unheated storage facility) as the control, and the other involving in situ soil burial at 15 cm depth as the experimental treatment. By integrating seed size, we explored the differential effects of in situ burial on seed germination and survival across species, as well as the underlying mechanisms. We propose the following hypothesis: small-seeded species are better adapted to burial conditions in terms of germination and survival, whereas large-seeded species are better adapted to non-burial conditions. This reflects the reproductive ecological strategies developed by plants through long-term adaptation to their specific environmental conditions. The findings of this study contribute to a deeper understanding of the ecological adaptation strategies in seed reproduction of subalpine wet meadow plants, play a significant role in revealing the formation mechanisms of soil seed banks, and provide a scientific basis for the informed conservation, restoration, and management of subalpine wet meadows.

## 2. Materials and Methods

### 2.1. Study Area

Plant seeds for this experiment were collected and buried at Napahai Wetland (99°37′–99°43′ E, 27°49′–27°55′ N), an internationally significant lake-marsh ecosystem in northwestern Yunnan, China. Situated at an average elevation of 3260 m, this representative seasonal karst alpine wetland features a climate characterized by low temperatures, alpine conditions, prolonged frost periods, and pronounced drought during winter-spring. The site records an annual mean temperature of 5.4 °C, with average temperatures of −3.8 °C in the coldest month and 13.2 °C in the warmest month. The Napahai Wetland watershed lies within a cold-temperate plateau monsoon climate zone, characterized by prevailing southerly and south-southwesterly winds year-round. The multi-year average precipitation is approximately 658 mm, with distinct dry and wet seasons: the rainy season extends from May to October, while a pronounced dry season occurs from November to April, marked by scanty rainfall and severe drought.

### 2.2. Seed Collection and Processing

Seeds for this experiment were collected during the maturation period (July–September 2023) from species listed in Table 1. For each species, seeds were collected from a minimum of 15 spatially distinct sampling points, with each point providing 10–20 individuals according to per-plant seed yield. Sampling points were separated by ≥10 m to ensure population representativeness and adequate seed quantities. Freshly collected seeds, predominantly retaining husks or glumes, were air-dried in ventilated shade for several days prior to husk removal. Following thorough homogenization, seeds were packaged in permeable bags and stored in a dark, low-humidity storage room at ambient temperature for subsequent use.

After processing, seeds of each species were thoroughly mixed, and plump, healthy seeds were randomly selected for the experiment. The 100-seed mass was determined with a 0.0001 g electronic balance, with five replicates per species. Prior to field experiment initiation, three replicates per species were established, each comprising 50 seeds subjected to tetrazolium viability testing. The viable seed proportion was calculated for each species. Subsequent calculations of germination and mortality rates for all treatments were corrected based on these viability data.

### 2.3. In Situ Burial Experiment

The experiment included two treatments: in situ burial and a control. For each species under each treatment, eight replicates were established. Each replicate consisted of one nylon mesh bag containing 30 healthy, plump seeds. A total of 192 nylon mesh bags were used for the 12 species. All seeds were deployed in early December 2023 at the experimental site in the Napahai wet meadow, Yunnan Province. Burial treatment group: The eight replicates for each species were buried at eight distinct locations, with a minimum inter-point distance of 10 m to ensure spatial independence, and at a uniform depth of 15 cm. This depth was selected based on the vertical distribution characteristics of the soil seed bank, namely that the majority of the seed bank exists in the upper 15 cm of soil, while density is very low below this depth [23,24]. Therefore, this depth effectively simulates the burial conditions that seeds are likely to encounter in natural settings. Control treatment group: The additional eight replicates were placed in a corrugated steel shed near the burial site. The shed was maintained without any heating facilities—a common seed storage condition that can approximately simulate the vernalization process seeds experience under natural conditions. In early August 2024, all seed bags were retrieved and transported back to the laboratory. Subsequently, four replicates each were randomly selected from both the control and burial treatment groups for every species to determine germination and mortality rates.

### 2.4. Determination of Seed Germination Rate

In the laboratory, four of the eight seed replicates per species from both the burial and control treatments were randomly selected. Seeds were rinsed with distilled water to remove decayed and incomplete specimens. The sound seeds were then placed in 9 cm-diameter Petri dishes lined with two layers of filter paper, moistened with distilled water to saturation, and incubated in incubator (MGC-450HP-2, Shanghai Yiheng Technology Co., Ltd., Shanghai, China). The incubator was set to 20 °C/10 °C (day/night) with a 14 h light/10 h dark photoperiod. Daily protocols included monitoring Petri dishes for moisture deficiency, replenishing distilled water to maintain saturation, and removing germinated seeds. The germination trial terminated after 45 days when no additional germination occurred for seven consecutive days.

### 2.5. Determination of Seed Mortality Rate

In the laboratory, the remaining four replicates of seeds from the burial treatment were taken. Seeds retrieved from burial were rinsed with distilled water, and decayed/missing seeds in each nylon mesh bag were recorded as the soil mortality count. The remaining seeds underwent tetrazolium viability testing to determine viable seed counts, thereby deriving mortality counts. These two components (decayed/missing seeds + post-test mortality) were summed to calculate final seed mortality rates for the burial treatment. The remaining four replicates from the control treatment were directly subjected to a tetrazolium test to assess seed viability. Based on the number of remaining viable seeds obtained, the number of dead seeds and the seed mortality rate under control conditions were determined.

### 2.6. Data Analysis

#### 2.6.1. Germination and Mortality Metrics: Calculation Methods

The following metrics were calculated to assess seed germination and mortality. The seed germination rate (GR) and seed mortality rate (MR) were defined as follows:

(1) $$GR\;\left(\%\right)=\left(\frac{G}{\mathrm{N\_v}iable}\right)\times 100\%\;$$

(2) $$MR\;\left(\%\right)=\left(\frac{D}{\mathrm{N\_t}otal}\right)\times 100\%\;$$

where G is the number of seeds that reached full germination in each replicate of a treatment, D is the number of dead seeds in each replicate of a treatment, N_viable is the total number of viable seeds in that replicate of the treatment, and N_total is the total number of seeds in the that replicate of the treatment.

To evaluate the effect of burial compared to the control, the relative changes in germination (RCG) and mortality (RCM) were calculated as:

(3) $$RCG\;\left(\%\right)=\left[\frac{\left(\mathrm{G\_b}urial-\mathrm{G\_c}ontrol\right)}{\mathrm{G\_c}ontrol}\right]\times 100\%\;$$

(4) $$RCM\;\left(\%\right)=\left[\frac{\left(\mathrm{D\_b}urial-\mathrm{D\_c}ontrol\right)}{\mathrm{D\_c}ontrol}\right]\times 100\%\;$$

where G_burial is the number of germinated seeds after burial, G_control is the number of germinated seeds in control, D_burial is the number of dead seeds after burial, and D_control is the number of dead seeds in control.

*Data correction*: Both germination and mortality rates were corrected based on the viable seeds rate. Corrected germination rates exceeding 100% were capped at 100% to align with biological reality.

#### 2.6.2. Statistical Processing and Analysis

First, we performed a one-way analysis of variance (ANOVA) to examine whether there were significant differences in germination and mortality rates among the 12 plant species under different treatments. Subsequently, paired-sample *t*-tests were conducted to compare germination and mortality rates between control and in situ burial treatments for each species, determining whether significant differences existed between treatments. Additionally, a paired-sample *t*-test was employed to determine whether there was a significant difference between the mean germination and mortality rates of the 12 species under burial treatment compared to those in the control group. We constructed a correlation network analysis, calculated Pearson’s correlation coefficients (r) between seed size and various germination/mortality metrics, and assessed the statistical significance of each correlation. Principal component analysis (PCA) was performed on seed size along with various metrics for germination rate and mortality, based on different burial response types. One-way ANOVA was employed to analyze the significance of differences in seed size among the various burial germination types and survival types, followed by post hoc testing using the least significant difference (LSD) method. A general linear model was applied to examine the linear relationships between seed size and burial germination rate, control mortality rate (CK mortality), and the rate of mortality change (δmortality). All statistical analyses were performed in the R statistical environment (version 4.3.1). One-way ANOVA and LSD post hoc tests were conducted using the ‘agricolae’ package. Paired *t*-tests (two-tailed) were performed using the t.test() function. Correlation network analysis was carried out with the “Hmisc” and “linkET” packages. PCA was implemented using the “vegan” package. General linear models were built using the lm() function. All figures were generated using the ‘ggplot2’ package.

## 3. Results

### 3.1. The Effect of Burial on Seed Germination Rate

**Figure 1:**
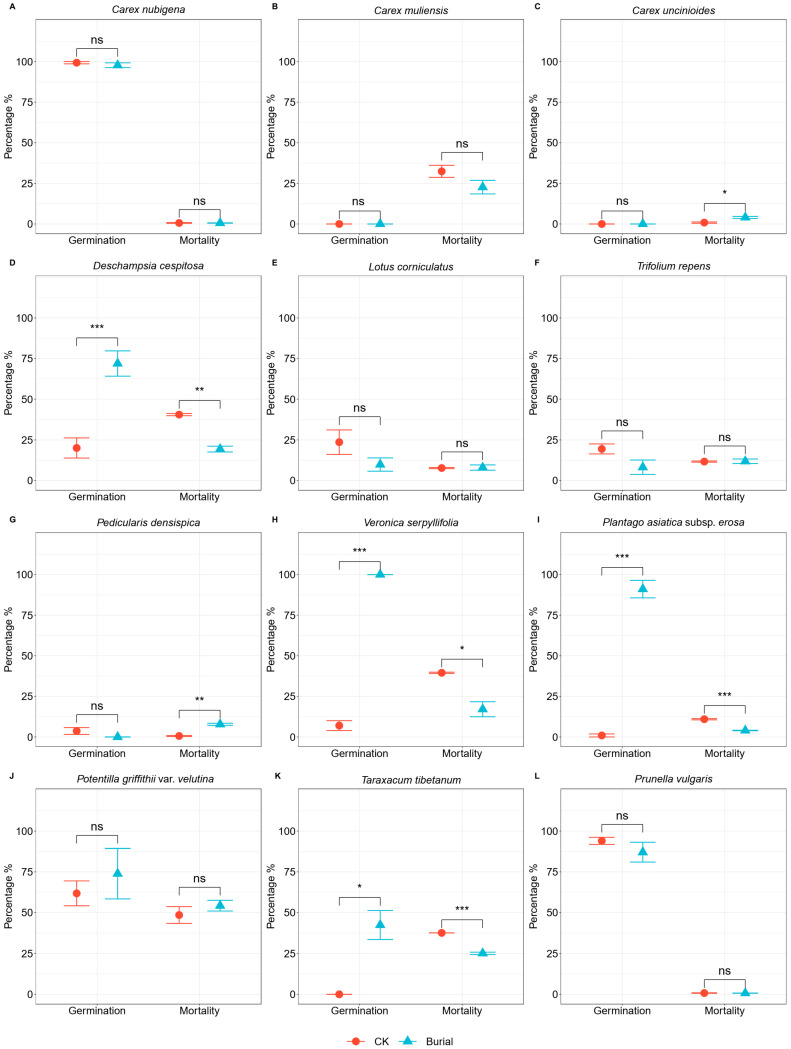
Germination and mortality rates of seeds of 12 plant species from the Napahai wet meadow. Subplots (**A**–**L**) represent comparisons of germination percentage and mortality under control and burial treatments for each of the 12 species, respectively. Significant differences were determined by paired *t*-tests: ns, no significant difference, * *p* < 0.05, ** *p* < 0.01, *** *p* < 0.001. Error bars represent standard errors. CK: control treatment; Burial: in situ burial treatment.

One-way ANOVA revealed highly significant differences in germination rates among the 12 plant species under both control and in situ burial treatments (Control: F = 88.86, *p* < 0.001; Burial: F = 44.72, *p* < 0.001). Under control treatment, the average germination rate across the 12 species was 27.49%, ranging from 0% (in *Carex muliensis*, *Carex uncinioides*, and *Taraxacum tibetanum*) to 100% (in *Carex nubigena*). Under control conditions, two plant species (16.67% of total) displayed germination rates between 75% and 100%, one species (8.33%) exhibited germination between 50% and 75%, and nine species (75%) recorded germination below 25%. Among the latter group, three species (25% of total) showed no germination (Figure 1). Under in situ burial treatment, the average germination rate across the 12 species was 48.51%, ranging from 0% (in *Carex muliensis*, *Carex uncinioides*, and *Pedicularis densispica*) to 100% (in *Veronica serpyllifolia*). Under burial treatment, four plant species (33.33% of total) exhibited germination rates between 75% and 100%, two species (16.67%) showed germination between 50% and 75%, and one species (8.33%) demonstrated germination between 25% and 50%. Five species (41.67%) had germination rates below 25%, among which three species (25% of total) displayed no germination (Figure 1). Paired *t*-tests comparing mean germination rates of the 12 species between control and in situ burial treatments indicated no statistically significant alteration in overall germination rates (t = 1.69, *p* = 0.119).

The burial treatment significantly enhanced seed germination rate in *Deschampsia cespitosa* (*p* < 0.001), *Veronica serpyllifolia* (*p* < 0.001), *Plantago asiatica* subsp. *erosa* (*p* < 0.001), and *Taraxacum tibetanum* (*p* < 0.05), with increases of 51.89%, 93.00%, 90.11%, and 42.43%, respectively, compared to control. For the remaining eight species, burial exerted no significant effect on germination rate.

### 3.2. The Effect of Burial on Seed Mortality Rate

One-way ANOVA revealed highly significant differences in mortality rates among the 12 plant species under both control and in situ burial treatments (Control: F = 101.62, *p* < 0.001; Burial: F = 46.14, *p* < 0.001). Under control treatment, the average mortality rate across 12 species was 19.31%, ranging from 0.60% (in *Pedicularis densispica*) to 48.55% (in *Potentilla griffithii* var. *velutina*). Under control conditions, seed mortality rates between 25% and 50% occurred in five plant species (41.67% of total), while seven species (58.33%) exhibited mortality below 25% (Figure 1). Under in situ burial treatment, the average mortality rate across the 12 species was 14.62%, ranging from 0.59% (in *Carex nubigena*) to 54.26% (in *Potentilla griffithii* var. *velutina*). Under burial treatment, one plant species (8.33% of total) displayed seed mortality between 50% and 75%, one species (8.33%) exhibited mortality between 25% and 50%, and ten species (83.33%) showed mortality below 25% (Figure 1). Paired *t*-tests comparing mean mortality rates across the 12 species between control and in situ burial treatments revealed no significant alteration in seed mortality (t = −0.93, *p* = 0.371).

The burial treatment significantly increased seed mortality in *Carex uncinioides* (*p* < 0.05) and *Pedicularis densispica* (*p* < 0.01), with increments of 3.20% and 7.21%, respectively, compared to control. Conversely, burial significantly reduced mortality in *Deschampsia cespitosa* (*p* < 0.01), *Veronica serpyllifolia* (*p* < 0.05), *Plantago asiatica* subsp. *erosa* (*p* < 0.001), and *Taraxacum tibetanum* (*p* < 0.001), showing decreases of 21.10%, 22.44%, 6.97%, and 12.54%, respectively. For the remaining six species, burial exerted no significant effect on seed mortality.

### 3.3. Relationships Between Seed Size and Germination/Mortality Rates Under Control and In Situ Burial Treatments

**Figure 2:**
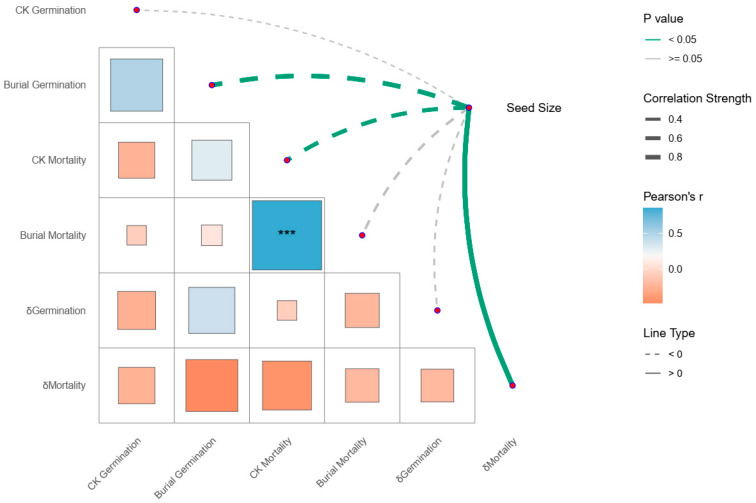
Correlation network heatmap between seed size and germination rate, mortality rate, and their relative rates of change under different treatments across 12 species. *** *p* < 0.001.

A correlation network analysis was constructed between seed size and: (1) germination and mortality rates in control groups, (2) germination and mortality rates in burial groups, and (3) relative change rates of germination and mortality between treatments. Results are presented in Figure 2. Results demonstrated that seed size exhibited a highly significant negative correlation with germination rate in burial groups (*p* < 0.01), a significant negative correlation with mortality rate in control groups (*p* < 0.05), and a highly significant positive correlation with the relative change rate of mortality (*p* < 0.01).

The results of PCA indicated that seed size made an important contribution to the classification of response types (Figure S1, Table S2). The first principal component axis (PC1) mainly determines the division of different response types, and seed size has a high contribution to PC1. One-way analysis of variance (Figure S2) revealed that although not all pairwise comparisons between response types showed statistically significant differences in seed size, clear differentiation was observed in mean values, suggesting a close relationship between the classification and seed size. These findings suggest that the promoting effects of burial treatment on seed germination and survival may tend to benefit smaller-seeded types.

## 4. Discussion

### 4.1. Species—Specific Response Mechanisms

Based on changes in germination and mortality rates after burial, this study categorized the 12 species into distinct response types, a classification that aligns with the frameworks proposed by Li et al. [14] and Zhang et al. [25]. According to germination responses, the species were classified into two types: burial-promoted germination (including *Deschampsia cespitosa*, *Veronica serpyllifolia*, *Plantago asiatica* subsp. *erosa*, and *Taraxacum tibetanum*) and burial-insensitive germination (the remaining eight species). Based on mortality responses, the species were categorized into three types: survival-inhibited (including *Carex uncinioides* and *Pedicularis densispica*), survival-promoted (including *Deschampsia cespitosa*, *Veronica serpyllifolia*, *Plantago asiatica* subsp. *erosa*, and *Taraxacum tibetanum*), and survival-insensitive (the remaining six species). Notably, *Deschampsia cespitosa*, *Veronica serpyllifolia*, *Plantago asiatica* subsp. *erosa*, and *Taraxacum tibetanum* exhibited simultaneous significant increases in both germination and survival rates following burial, categorizing them as “dual-promoted” species. First, burial was found to significantly reduce mortality rates in these species, suggesting their ability to effectively utilize the soil environment to avoid surface-level stresses (such as extreme temperatures and desiccation), thereby maintaining viability in the soil for extended periods. This high viability suggests their potential to maintain high density within the soil seed bank, serving as a critical seed source reserve within the plant community. Secondly, the burial treatment simulated the natural process of seeds temporarily entering the soil, providing them with a dark, thermally stable, and humid microenvironment. This not only effectively protects seeds from surface-level extreme climates and predator pressures [26], but may also significantly enhance germination rates by breaking physiological dormancy. For instance: *Veronica serpyllifolia* exhibited a remarkable increase in germination from 7% to 100% after burial; *Plantago asiatica* subsp. *erosa* showed a dramatic rise from 0.93% to 92.35%; *Deschampsia cespitosa* increased from 20.05% to 71.93%; *Taraxacum tibetanum* improved from 0% to 42.43%. Once environmental conditions become suitable (e.g., when disturbances create bare ground), these seeds can rapidly exit dormancy and germinate efficiently [27], demonstrating typical pioneer species characteristics [28]. Disturbances (e.g., animal digging or human activities) not only re-expose them to the surface soil [29,30], but also provide critical germination cues such as light signals and temperature fluctuations, enabling them to rapidly colonize resource-rich patches and facilitate community regeneration [31]. Therefore, burial can be regarded as a germination enhancement technique that simulates natural processes, demonstrating significant application potential for improving the germination performance of specific species in vegetation restoration efforts.

In contrast, for those species exhibiting consistently low germination rates under both control and burial conditions (such as *Carex mulensis*, *Carex uncinioides*, and *Pedicularis densispica*), we hypothesize that they adopt a “bet–hedging” or risk-spreading strategy in germination timing [32]. This strategy involves maintaining dormancy and staggering germination to cope with environmental uncertainty. Meanwhile, certain species (such as *Carex nubigena* and *Prunella vulgaris*) exhibited high germination rates regardless of burial treatment, indicating that burial had neither a significant promoting nor inhibiting effect on their germination. Different species have developed distinct ecological adaptation strategies in response to burial, and these differences likely originate from variations in seed traits (such as seed coat structure and dormancy type) and sensitivity to environmental signals (light, temperature, moisture)—a finding that has been precisely demonstrated in studies by Wang et al. [33] and Peng et al. [34]. Therefore, when utilizing seed propagation for vegetation restoration or reconstruction, species-specific responses must be given full consideration, and tailored seed treatment and management strategies should be developed for different species. This study demonstrates that simulated natural burial treatment is effective for the four species characterized by “dual-promoted” traits. This method can be utilized to enhance their seed survival and germination performance, thereby supporting vegetation restoration efforts.

### 4.2. Relationships Between Seed Size and Response of Germination and Mortality Rates

Seed size is a key and relatively stable trait in plant life history, which not only reflects the amount of nutrients stored within the seed but also serves as the material basis for seed emergence and seedling establishment [19,35]. This study found that under non-burial (control) conditions, larger seeds exhibited lower mortality rates, confirming predictions of the seed size evolutionary trade-off theory [36]. Due to their richer nutrient reserves and more developed protective seed coat structures, larger seeds demonstrate enhanced resistance to surface drought, temperature fluctuations, and microbial infestation [37,38]. This pattern has also been widely verified in grassland and desert ecosystems [39,40]. Under burial conditions, smaller seeds demonstrated higher germination rates and exhibited a relatively small increase in mortality rate, indicating their better adaptation to the soil burial environment. Due to their small size and light mass, smaller seeds can more easily enter the soil to avoid surface stresses, thereby forming a persistent seed bank [34,41]; meanwhile, they generally possess a higher embryo ratio and more sensitive environmental response mechanisms, enabling rapid germination under suitable conditions [33]. Conversely, while larger seeds exhibit strong stress resistance on the soil surface, their physical structure and germination characteristics may limit their adaptability to burial environments [42]. Collectively, these findings support the initial hypothesis of this study, fundamentally confirming that smaller seeds are better adapted to burial conditions while larger seeds are more suited to non-burial environments.

Although not all pairwise comparisons between different response types showed statistically significant differences, the results from both PCA and ANOVA (Figures S1 and S2; Table S2) nevertheless reveal a substantial contributory role of seed size in the classification. This indicates that seed size, as a key functional trait, participates in regulating the coordinated adaptation between germination and survival. This size-dependent response represents an ecological strategy evolved through long-term natural selection: smaller seeds, due to their physical properties, easily penetrate the soil environment and have evolved burial-dependent, rapid-response germination behaviors through prolonged adaptation; whereas larger seeds have adapted to persist and germinate in surface conditions by allocating resources to seed mass and defensive structures. This study, starting from seed size, provides important insights for understanding the formation mechanisms of soil seed banks and for developing or selecting seed propagation techniques in vegetation restoration.

### 4.3. Study Limitations and Future Directions

First, this study employed a short-term burial duration covering the winter and part of the growing season. Although this period plays a critical role in seed dormancy release, early germination, and survival [43], and can effectively reflect the initial impact of burial on seed fate, it still has temporal limitations by failing to capture the persistent changes in seeds over multiple years of burial. Therefore, future research should employ burial experiments conducted over multiple consecutive years (e.g., 1–3 years) to systematically investigate the long-term responses of seed survival and germination to burial. Such research would be more conducive to revealing the formation and maintenance mechanisms of persistent seed banks. It would particularly enhance our understanding of the germination strategies and population regeneration patterns of species with complex dormancy traits, thus carrying significant implications for comprehending soil seed bank dynamics and long-term vegetation restoration potential.

Second, this study employed a burial depth of 15 cm. This depth was selected based on the typical distribution of seed banks within the upper 15 cm of soil [23,24]. Choosing this critical point helps simulate the typical burial environment that seeds may encounter under natural conditions. However, seed burial depth is highly variable under field conditions. Soil physicochemical properties—including temperature, moisture, aeration, and microbial activity—vary significantly across different depths, thus potentially exerting distinct effects on seed germination and mortality [26,44]. Therefore, future research could conduct burial experiments by subdividing the 0–15 cm soil layer (e.g., 0–5 cm, 5–10 cm, 10–15 cm) to systematically evaluate the effects of depth on seed behavior. Determining the optimal burial depth for different species will not only contribute to a deeper understanding of the formation and maintenance mechanisms of soil seed banks, but also holds significant practical implications for developing targeted seed germination enhancement techniques and improving the efficiency of seed utilization in vegetation restoration practices.

Additionally, the control treatment in this study involved storage under ambient conditions without heating facilities near the burial site (in situ), which represents only one common form of seed storage. Although this control provides a comparable benchmark for assessing burial effects, it still has limitations: it represents only one of many possible storage methods and cannot fully depict the spectrum of actual microenvironments that seeds may encounter in natural settings. Thus, the assessment of burial effects based on a single control has certain limitations. Future studies should systematically incorporate more diverse storage conditions, particularly treatments that closely simulate natural states, such as surface placement, storage on simulated standing dead plant material, and indoor storage with controlled temperature and humidity. By comparing seed germination and survival responses across multiple storage conditions, we can not only more comprehensively reveal the physio-ecological mechanisms of species adaptation to their environments, but also especially help discern the response specificities of different species to their particular storage microenvironments. Such comparative studies will further inform the optimization of species-specific seed storage technologies and germination enhancement protocols.

## 5. Conclusions

The responses of seed survival and germination to in situ soil burial in common species of subalpine wet meadows are species-specific. Burial is beneficial for the survival and germination of smaller-seeded species but detrimental to larger-seeded ones, reflecting the ecological strategies formed through plants’ long-term adaptation to their environment based on seed traits. The findings contribute to our understanding of seed bank formation mechanisms and vegetation succession patterns following disturbance, while also providing practical guidance for the development of germination-promotion techniques in vegetation restoration practices. Simulating the specific environmental conditions that mature seeds with varying traits encounter or experience after dispersal may provide valuable clues for exploring optimal storage conditions for specific species to enhance survival and promote germination. We can emulate nature by conducting more detailed experiments based on this clue to explore optimal conditions for enhancing seed survival and germination, thereby providing technical support for vegetation restoration.

## Figures and Tables

**Table 1:** Species and seed size of 12 common plants in Napahai Wet Meadow.

Species Name	Abbreviation	Family Name	Seed Mass/mg
*Carex nubigena*	*C. nub*	Cyperaceae	0.35 ± 0.01
*Carex muliensis*	*C. mul*	Cyperaceae	0.69 ± 0.01
*Carex uncinioides*	*C. unc*	Cyperaceae	0.88 ± 0.02
*Deschampsia cespitosa*	*D. ces*	Gramineae	0.20 ± 0.01
*Lotus corniculatus*	*L. cor*	Leguminosae	0.93 ± 0.01
*Trifolium repens*	*T. rep*	Leguminosae	0.49 ± 0.02
*Pedicularis densispica*	*P. den*	Scrophulariaceae	1.49 ± 0.03
*Veronica serpyllifolia*	*V. ser*	Scrophulariaceae	0.05 ± 0.00
*Plantago asiatica* subsp. *erosa*	*P. asi*	Plantaginaceae	0.42 ± 0.01
*Potentilla griffithii* var. *velutina*	*P. grif*	Rosaceae	0.37 ± 0.00
*Taraxacum tibetanum*	*T.* *tib*	Compositae	0.51 ± 0.01
*Prunella vulgaris*	*P. vul*	Labiatae	0.63 ± 0.01

Note: Seed mass per seed values in the table represent mean ± standard error.

## Data Availability

Data are contained within the article and Supplementary Materials.

## Supplementary Materials

The following supporting information can be downloaded at https://www.mdpi.com/article/10.3390/plants14192975/s1. Figure S1: Principal component analysis of seed size and various metrics based on burial response types for seed germination and survival. Figure S2: Comparison of seed size differences among types of burial responses in seed germination and survival. Figure S3: Linear regression analysis diagram of the seed size of 12 species and their buried germination rate (A), control mortality rate (B), and mortality change rate (C). Table S1: Number of germinated and dead seeds of 12 plant species under two treatments, along with the average values of corrected germination rate and mortality rate for each treatment. Table S2: Factor loadings of principal component analysis for different burial types (germination type and survival type).
