# Supplementary material for: Response Patterns and Mechanisms of Seed Germination and Mortality of Common Plants in Subalpine Wet Meadows to In Situ Burial
Source: Plants (Basel). 2025 Sep 25;14(19):2975. doi: 10.3390/plants14192975 (PMC12525858; doi:10.3390/plants14192975)
Supplement: Supplementary file 1 [file plants-14-02975-s001.zip › plants-3849344-supplementary.pdf]

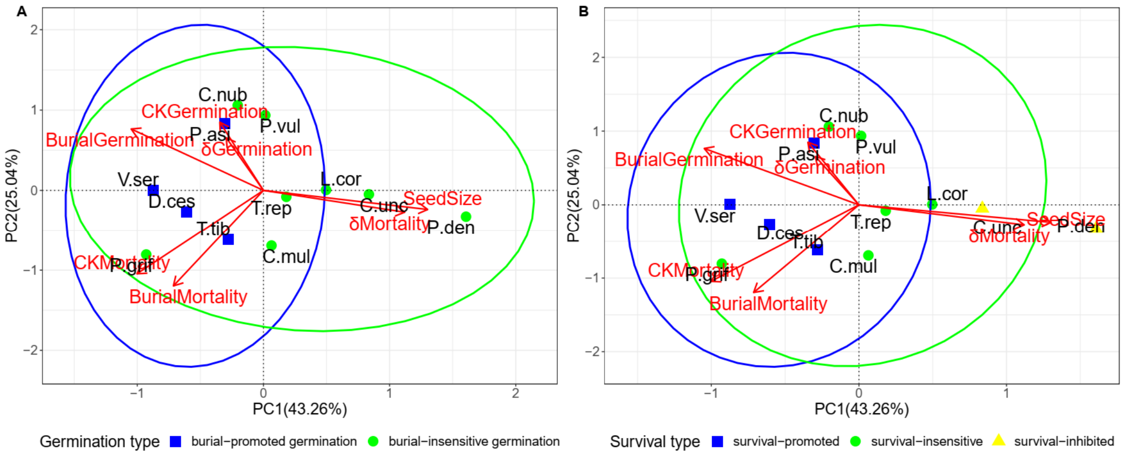

Figure S1. Principal component analysis of seed size and various metrics based on burial response types for seed germination and survival

Note: The abbreviations for species names are the same as those in Table 1. In subfigure A, the blue square points represent the burial-promoted germination type, indicating species that showed a significant increase in germination rate after burial; the green circular points represent the burial-insensitive germination type, denoting species with no significant change in germination rate following burial. In subfigure B, the blue square points correspond to the survival-promoted type, representing species that exhibited a significant decrease in mortality rate after burial; the green circular points indicate the survival-insensitive type, referring to species with no significant change in post-burial mortality rate; while the yellow triangular points depict the survival-inhibited type, characterizing species that demonstrated a significant increase in mortality rate following burial.

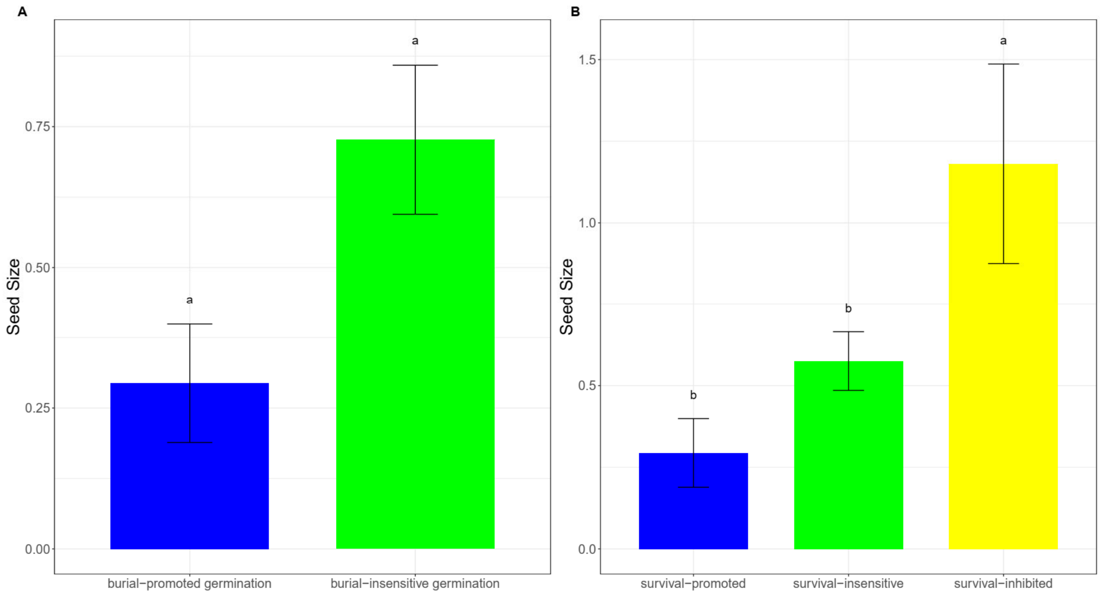

Figure S2. Comparison of seed size differences among types of burial responses in seed germination and survival

Note: Different letters indicate significant differences ($P < 0.05$). In subfigure A, blue bars represent seed sizes of the burial-promoted germination type, indicating species that showed a significant increase in germination rate after burial; green bars represent seed sizes of the burial-insensitive germination type, denoting species with no significant change in germination rate following burial. In subfigure B, blue bars indicate seed sizes of the survival-promoted type, representing species that exhibited a significant decrease in mortality rate after burial; green bars represent seed sizes of the survival-insensitive type, referring to species with no significant change in post-burial mortality rate; yellow bars depict seed sizes of the survival-inhibited type, characterizing species that demonstrated a significant increase in mortality rate following burial.

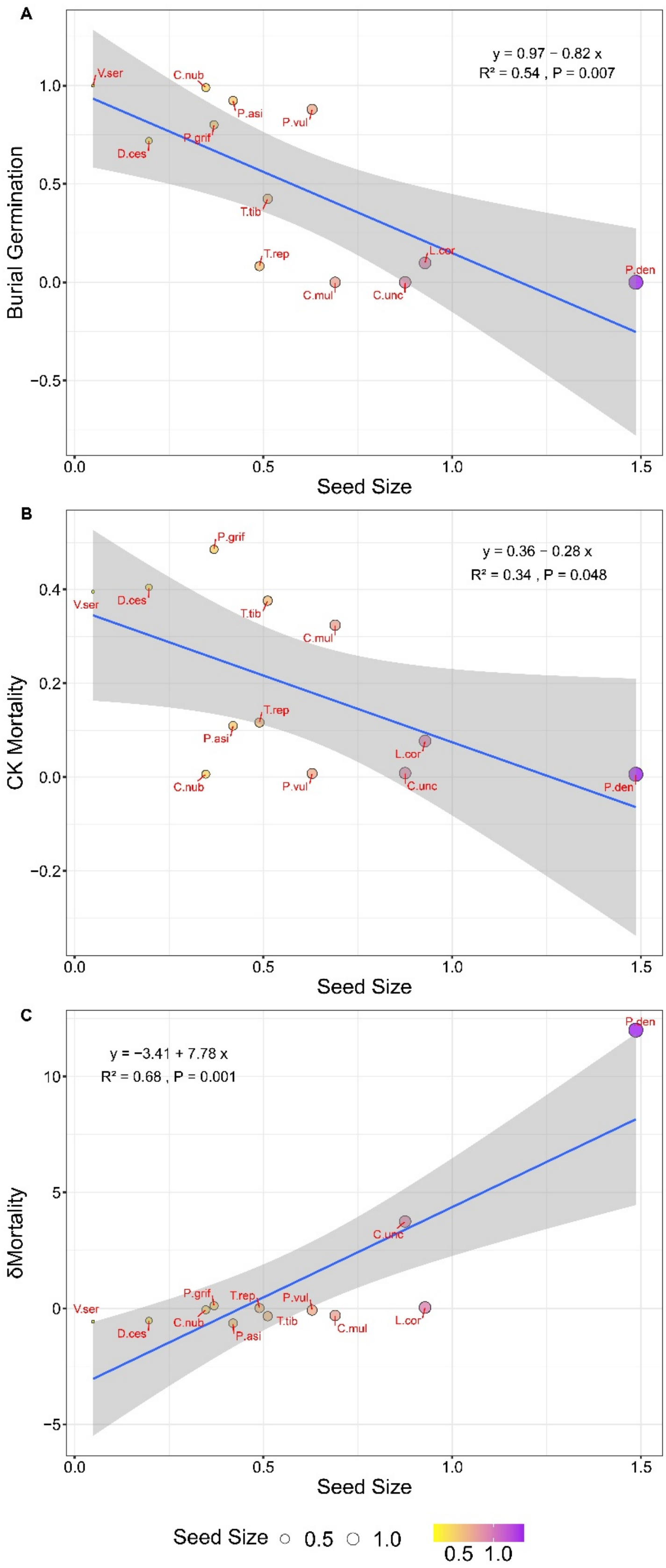

Figure S3. Linear regression analysis diagram of the seed size of 12 species and their buried germination rate (A), control mortality rate (B), and mortality change rate (C)

Note: The abbreviations for species names are the same as those in Table 1.

Table S1. Number of germinated and dead seeds of 12 plant species under two treatments, along with the average values of corrected germination rate and mortality rate for each treatment.

Species	Germination number		Mortality number		Viable seeds rate%	CK Germination% (after correction)	Burial Germination% (after correction)	CK Mortality% (after correction)	Burial Mortality% (after correction)
	CK	Burial	CK	Burial					
C.nub	29.25	28.5	4.5	4.25	96	100	98.96	0.63	0.59
C.mul	0	0	25	17.5	72	0	0	32.41	22.69
C.unc	0	0	2	9.5	89	0	0	0.85	4.05
D.ces	4.25	15.25	29.25	14	71	20.05	71.93	40.47	19.37
L.cor	6	2.5	12.75	13.25	85	23.62	9.84	7.70	8.00
T.rep	4.75	2	15.25	15.5	81	19.47	8.20	11.67	11.86
P.den	1	0	1.75	22.75	91	3.68	0	0.60	7.81
V.ser	1.5	26.25	29.5	12.75	71	7.01	100	39.52	17.08
P.asi	0.25	24.75	27.5	10	89	0.93	92.35	10.95	3.98
P.grif	11	14.25	21.25	23.75	59	61.80	80.06	48.55	54.26
T.tib	0	9.25	30	20	73	0	42.43	37.61	25.08
P.vul	27.25	25.5	6.75	6.25	97	93.97	87.93	0.78	0.72

Note: The abbreviations for species names are the same as those in Table 1.
Correction formula: Germination rate = Number of germinated seeds / (30 * Viable seeds rate)
Mortality rate = [Number of dead seeds * (1 - Viable seeds rate)] / (30 * Viable seeds rate)

Table S2. Factor loadings of principal component analysis for different burial types (Germination type and survival type)

Indicator	PC1	PC2
Seed Size	1.3021	-0.2378
CK Germination	-0.3459	0.8493
Burial Germination	-1.0484	0.7644
CK Mortality	-1.0031	-1.0455
Burial Mortality	-0.7137	-1.1966
δGermination	-0.2953	0.6558
δMortality	1.1202	-0.2717
